# Abstracts from FEDELAT 2016 meeting: Latin American Federation of IASP chapters

**DOI:** 10.1097/PR9.0000000000000568

**Published:** 2016-08-09

**Authors:** Alfredo Covarrubias-Gómez, Elias Atencio

We are pleased to share the abstracts of presentations from the recent XI Congreso Latinoamericano de dolor FEDELAT, June 15 to 17, 2016 in Costa Rica. The Latin American Federation of IASP Chapters (FEDELAT, in Spanish) was founded in 1990 by the presidents of the Mexican, Colombian, Argentinian, and Puerto Rico's IASP Chapters. To date FEDELAT is composed of 18 IASP chapters. The main purpose of the Federation is to encourage education and networking relations among chapters strengthening regional collaboration.

Over the last 14 years FEDELAT has maintained a close relation with the Spanish and Portuguese IASP chapters. Together, the 3 institutions, conducted the Ibero-American Reunion (RIAD, in Spanish). This event takes place every year, gathers professionals from both Europe and Latin-America, and exposes the regional advances on diverse pain issues.

FEDELAT had an important collaboration in events that promote education in Latin America. The former president, Dr Fabián Piedimonte, from both FEDELAT and the Argentinian IASP chapter represented the regional committee that organized the IASP World Pain Congress in Buenos Aires. FEDELAT's president is Dr Elias Atencio. Dr Atencio and the directive board have continued this work and over the last 2 years had organised the following events:

(1) The Hands-On Course on interventional techniques for pain control which took place in Buenos Aires (Argentina) from April 20 to 30, 2016; another will take place in Panama City (Panama) from November 10 to 20, 2016.
(2) The Annual RIAD occurred in Sevilla, Spain in 2015 and in San Jose, Costa Rica in 2016. In Costa Rica most lectures were centered on “articular pain” according to the IASP International Day Against Pain.
(3) FEDELAT is collaborating with the Mexican IASP Chapter to promote and organize the IASP Pain Management Camp. This second event in Latin America will include lectures about articular pain, cancer pain, acute pain, and opioids pharmacology and will take place October 10 to 15, 2016 in Mexico City, Mexico.
(4) Also the federation is developing research protocols to assess epidemiological aspects of chronic pain and conducted Practice Guidelines adapted to Latin American needs. These guidelines were published in a peer reviewed medical journal indexed in LILIACS.

During the Annual RIAD 4 general modules were discussed cancer pain and palliative care, research and education to improve pain curricula in Latin America, pharmacological pain relief, and interventional techniques for pain relief. In conclusion, the Federation and regional IASP Chapters are working together, harder than ever, in order to achieve a Latin America Without Pain.

Alfredo Covarrubias-Gómez, MD

Pain and Palliative Medicine

http://www.covarrubias-gomez.org

Federación Latinoamericana de Asociaciones para el Estudio del Dolor (FEDELAT)

Latin-American Federation of Associations for the Study of Pain

Secretary

Mexican Association for the Study and Treatment of Pain: An International Association for the Study of Pain Chapter

Past president

National Centre for the Study of Pain

CEO and founder

Department of Pain and Palliative Medicine, Mexican NHI on Internal Medicine

Attending physician/Associate professor

**Acknowledgement:** We thank Dr Melissa Garcia-Pons for her invaluable help with translation of some of the abstracts.

## Abstracts

### Cancer related pain: an update

Alfredo Covarrubias-Gomez, MD

Department of Pain and Palliative Medicine at the National Institute of Internal Medicine, Mexican NIH.

It is being reported that about 50% of cancer patients will report pain. Opioid therapy has been considered safe and effective in the management of moderate to severe pain intensity in cancer patients.

Morphine Consumption Data has been considered as an indicator to assess the efficacy of cancer pain treatment. Worldwide opioid consumption shows a significant disparity. Average opioid consumption in Latin-American countries is about 6 mgME “*per cápita*” (mgME, milligrams in morphine equivalence) while in Canada and USA report an average of 722.7 mgME and 717.8 mgME “*per cápita*” respectively.

Opioid therapy for cancer-related pain management poses 3 major issues: (1) limited opioid use promoting an inefficient pain control, (2) rightful concerns about inappropriate opioid use even in the cancer population, and (3) presence of chronic pain in cancer survivors.

Pharmacological management includes:

(1) Non-steroid Anti-inflammatory Drugs (NSAIDs): These drugs had shown insufficient analgesia in moderate and severe pain intensity, an analgesic ceiling effect without dose-dependent relation, and a dose-dependent occurrence of adverse effects.
(2) Opioid therapy includes Fentanyl, Oxycodone, Tapentadol, Methadone, Buprenorphine, and Morphine. These drugs are effective and safe for cancer-related pain management. However some of them pose issues related to the strength of the evidence (Methadone and Tapentadol). Transcutaneous Fentanyl had been studied in at least 4 meta-analyses for breakthrough pain.

Non-pharmacological management includes:

(1) Neurolytic celiac plexus block: This approach reported a persistent benefit in a long-term follow-up beyond 3 months.
(2) Transcutaneous Electrical Nerve Stimulation (TENS): This technique failed to provide sufficient evidence to determine its effectiveness in cancer-related pain.
(3) Acupuncture: This approach failed to provide sufficient evidence to determine its effectiveness in cancer-related pain.
(4) Massage: Despite being weak, the available evidence suggests a beneficial effect of massage techniques for cancer pain relief.
(5) Traditional herbal medicine: This approach combined with conventional therapy might be efficacious as an adjunctive therapy for patients with cancer pain.

Pain in cancer survivors is observed in 30% to 60% of survivors. Although there is not any specific guideline opioids may be considered for moderate to severe pain that has been unresponsive to non-opioid therapies and non-pharmacologic approaches and when the chronic opioid therapy is likely to possess equivalent or better risk-to-benefit ratios.

## Ozone therapy and pain

Ana Pedro, MD

Hospital Professor Doutor Fernando Fonseca, Rua José Ferrão Castelo Branco, no. 1, Paço de Arcos, Lisboa, 2770-098, Portugal

Treatment with ozone is increasing worldwide as a result of its simplicity of application, high efficiency, good tolerance and almost no side effects. In 1785, Dr Martin van Marum, notes that a gaseus substance with characteristic odor and oxidative properties is produced as a result of a strong electrical discharge in the air. In 1840 Prof Cristian Schobein correlated the described changes with the formation of a gas called ozone. Composed by 3 oxygen atoms, the ozone is an unstable gas, denser and more soluble in water than oxygen. It is a powerful oxidant that can be produced by chemical electrolysis, electric discharge and ultra-violet radiation. Ozone cannot be stored and must be used immediately after its production because of its instability and rapid decomposition. At the concentrations used in clinical practice, ozone is believed to have immunomodulatory, anti-inflammatory, antibacterial, antiviral, fungicidal and analgesic characteristics. The application of ozone for treatment of pain is based on its potent analgesic and anti-inflammatory effect, resulting from decreased production of inflammatory mediators and oxidation (inactivation) of mediators of pain, improvement of microcirculation and tissue oxygenation, elimination of toxins and activation of endogenous analgesic mechanisms. Ozone therapy has several clinical indications in the area of ​​pain, especially in low back pain, herniated discs, disc-radicular disease and osteo-articular pathology. It can be applied locally or parenterally, and may be used more than one pathway in combination with a synergistic effect between them. Inhalation is contraindicated because it is highly toxic to the lungs. Further, direct intravenous injection of ozone is not recommended because of the risk of embolism. Ozone can be administered locally to intervertebral disc, paravertebral or epidural spaces, intramuscular, intraarticular, subcutaneous, in trigger points, vagina, bladder, urethra and rectum. Another way is parenteral auto-hemotherapy, consisting of a mixture of ozone and blood withdrawn from the patient by a specific device. Blood is then reinfused, without contact with the external environment.

## Medical use of cannabis and cannabinoids: evidence and controversy

Maria A. Rico, MD

Anestesiologist/Pain Specialist, Clinica Alemana Santiago, Chile

**Introduction:** In recent years there is a growing interest of patients and their families in the potential benefit derived from cannabis sativa use, to relieve cáncer related pain and other symptoms, and other chronic diseases. The existence of an endocannabinoids system and specific receptors are known since 1990.

**Mechanism of action:** Agonism of CB1 receptors modulate synaptic transmission of excitatory and inhibitory circuits. Effects depend on the specific neural area stimulated, acting upon analgesia, memory process, appetite, muscle tone, etc. Activation of CB2 receptors may influence the activity of cytokines in the immune response to inflammation.

**Scientific evidence of cannabinoids in medicine:** Nausea/vomiting in patients with cancer and chemotherapy, using synthetic derivatives as dronabinol and nabilone, showed significant benefit until year 2000. The introduction of antiemetic agents, such HT3 blockers, leave cannabinoids as a third line indication. The effect of increased appetite with THC derivatives is significant only in HIV patients; it has not been seen in cancer patients. Anxiolytic effects and improved night sleep, although they are popular, they are not support in the literature. Benefits in epilepsy and multiple sclerosis has been stated. The use in refractory epilepsy, is becoming very popular, but it is still anecdotal.

**Cannabinoids as analgesics:** Comparative studies of cannabinoids and opioids demonstrate an efficacy equivalent to a weak opioid as dihydrocodeine, with limitation of adverse effects in increasing doses (Dizziness, dry mouth, fatigue, drowsiness, euphoria, agitation).

**Non oncologic pain:** Systematic reviews show studies in a wide variety of pathologies and doses. Cannabinoids were administrated as natural and synthetic forms, via inhaled, oral and transmucosal route. The results are generally modest, with a relative greater efficacy in neuropathic pain, but always the benefits are limited by its non-negligible adverse effects when increasing doses. Then, the evidence of benefits is rather weak.

**In palliative care:** For pain control, it seems beneficial as an adjuvant to opioids treatment, allowing for reduction of their doses, and not as a single analgesic. It has also been pointed out that cannabinoids can contribute to a better quality of life, related to reduced anxiety, better night time sleep, appetite and are generally well appreciated by patients and families.

**Conclusion:** Cannabinoids in pharmaceutical forms could be useful in a selected group of patients, for the management of some symptoms.

## Indications for interventional techniques in refractory chronic pain management

Dr Marco Antonio Narváez Tamayo

Attending Specialist, Study and Treatment of Pain Unit at the Hospital Obrero No. 1 (Hospital materno-infantil), Caja Nacional de Salud, La Paz, Bolivia

**Introduction:** Minimally invasive percutaneous techniques have drastically changed the therapeutic paradigm of conventional surgery. Current training, skills, instruments, image accuracy among other factors have brought us forward, achieving access to what was unthinkable many years, so we now can inhibit, control or solve problems that detract greatly the quality of life of our patients with refractory chronic pain.

**Objective:** Determine the indications of interventional techniques in the refractory chronic pain management.

**Material and method:** A non-systematic review of the literature was conducted, identifying articles on the basis of the following MeSH terms: indications, interventional treatment, technologies, blockade, radiofrequency, refractory chronic pain or neurolysis. Those who presented more than 3 terms were included in the search. Considerations included place or site where the study has developed, and the number of cases reviewed.

Different techniques commonly used in specialized units of treatment pain were reviewed, selecting the most frequent and those enrolled with the strongest evidence available to date. These include thermal radiofrequency of the medial branch of dorsal roots, cervical epidural analgesia, percutaneous epidural neuroplasty with Catheter Racz, radiofrequency of the sacroiliac joint, vertebroplasty, kyphoplasty, cementing the hip in mode femoro-cementoplasty, disc nucleoplasty by radiofrequency, neurolysis of stellate ganglion, selective inhibition of the splanchnic nerves, celiac plexus neurolysis, superior hypogastric plexus and ganglion Walther (odd node), radiofrequency of the dorsal root ganglion, foraminoplasty, spinal infusion in chronic cancer pain, among the most studied.

**Results:** In order to specifically review the indications for minimally invasive treatment, they were classified into 4 major groups: infusion, modulation, radio frequency and neurolysis. In this regard we show the results of some of the most common techniques of this particular chapter. Analysis also included procedures like control or relief of chronic pain in patients undergoing bone cementingm, vertebral cementation and cementation of long bones (femur, humerus, tibia). A common denominator for most of minimally invasive procedures, has been the low level in the pain intensity during the procedure, as well as in the post-operative period. Nevertheless, the biggest difference is represented by the lower risk, low complication rate and substantially a minimum percentage of adverse effects in this research group.

**Conclusion:** The increasing development of minimally invasive techniques is proving in recent years, encouraging results in relation to the relief of chronic pain refractory to conservative measures. These techniques are safe when performed in specialized centers experience with interventional pain techniques and the necessary guidelines for the quality of the process are respected.

## Rational use of opioids

Alicia Alonso Cardaño, MD

Hospital Universitario de Leon, Altos de Nava S/N, Leon, Leon 24071, Spain

Opioid analgesics are effective tools for the treatment of moderate to severe pain of all individuals that are subject of the right and dignity of relief unnecessary suffering, but it is important to develop parallel tools to prevent and reduce the dramatic increase in opioid addiction behaviors. Comparison of opioid prescriptions in North America vs Europe might reveal factors involved in the so-called “opioid epidemic.” The main controversy is about opioids for pain management of non-oncological origin or benign processes, in which long term therapies might be relevant. Bibliographic review of recent medical and scientific literature most recent published in journals of impact, in order to establish the current situation, with the target set in the extraction of predisposing factors, possible existence of predictive factors, investigate different ways of dealing with opioid prescription and even different ways of addressing similar conditions in different continents. It was found that prescribing opioids may contribute to the development of a misuse of opioids, which has led to the development of clinical guidelines for recommendations and specific programs by various associations both medical and governmental, plus the manifestations promoted by the group of patients and their corresponding associations scattered across different states. In conclusions, strategies to minimize addiction to treatments seem to be effective, although a longer time to determine these observations analysis will be needed, while the controversy over the use or misuse of opioid prescription still will coexist in parallel. The selection of one type of drug or another to address the pain of nonmalignant origin should be considered in the field of each chronical pain syndrome in question, taking into account the concurrent diseases of each patient, contraindications relating to the treatment, the response that the individual has shown to pretreatments, its beneficial effects, and even the preferences in this regard that the patient can present having in mind other possibilities of treatment for his/her syndrome in particular, as are other nonpharmacological measures.

## Evidence in favor of neurolytic blocks for cancer pain

Prof João Batista Santos Garcia, MD, PhD,

Pain and Palliative Care Service- Cancer Hospital of Maranhao, São Luis, Brazil.

Pain is one of the most common symptoms of cancer, affecting over 90% of the patients during the course of their treatment. In advanced cases pain is referred to as severe in half the cases, and it originates from multiple factors. In order to address this issue, the World Health Organization (WHO) proposed an approach to the treatment of cancer pain patients using a 3 ladder stair system in which opioids are viewed as the cornerstone to the treatment, but also highlighting the importance of adjuvants. This strategy has been considered effective in controlling pain, reducing pain in 70% of patients, however, 30% of cancer patients still end up developing refractory pain. For these cases, interventional techniques have been proposed as a fourth step in the analgesic ladder, nonetheless pain specialists have considered this approach earlier in the course of treatment in order to lessen unnecessary suffering. Interventional therapy for cancer can be obtained through several techniques, including the injection of neurolytic substances. Neurolysis of the celiac plexus (NCPB) is often indicated for patients with pancreatic cancer with severe pain, although it may be helpful to other visceral tumors such as lower esophagus, gastric, hepatobiliary system, and intestinal to the proximal half of the transverse colon. NCPB improves pain, reduces opioid consumption, and reduces rates of side effects, including constipation, nausea, and vomiting, as compared with standard analgesic therapy. Neurolysis of the superior hypogastric plexus may be used to control pelvic cancer pain. And ganglion impar neurolysis is effective for rectal pain from rectal cancer or from radiotherapy complications (actinic rectitis). Intercostal nerve block is effective to manage pain arising from metastatic lesions to the ribs. One study showed that 80% of the patients noted greater than 50% improvement in pain and 56% reduced their analgesic use after the procedure. Nevertheless, evidence for those blocks is weak as it comes mainly from small case series or retrospective studies. Intrathecal neurolysis is a radical therapy indicated to patients suffering from perineal refractory pain. Since this procedure can produce significant morbidity, including bowel and/or bladder dysfunction, motor weakness, neuritis, and paresthesias, it is usually reserved for patients with low life expectancy and poor performance status. Although evidence for this neurolysis is favorable, it is also derived from poor quality studies. To conclude, interventional techniques may be used to promote better pain control for patients refractory to opioid therapy, or who are limited by side effects. Even so, since these procedures are invasive and are not free from treatment-related complications, clinicians must weigh possible benefits and risks before proceeding with the intervention.

## Nonsteroidal anti-inflammatory drugs: myths and realities

Patricia Gomez

Department of Anaesthesia and Pain, Colombian National University, Bogotá, Colombia

Their analgesic, and peripheral and central sensitization prevention actions make them the most formulated and used in self-medication drugs on a worldwide level. PubMed search of review articles with the keywords: Nonsteroidal anti-inflammatory & NSAIDs. **Several adverse events has been reported:** (1) **Gastrointestinal (GI):** these are one of the most common adverse events related to drugs. The relative risk of ulcers, perforations, and bleeding is of 5.3 higher in comparison to non-NSAID consumers. It is strongly recommended to identify GI risk factors and prophylaxis (including age above 60, presence of helicobacter, use of anti coagulants and other factors). The COX2 selective inhibitors are associated with the lower rate of GI complications and symptomatic ulcers. Nonetheless, the use of COX2 inhibitors can increase cardiovascular risks. (2) **Cardiovascular (CV):** NSAIDs use is associated with a little increase but consistent CV events like myocardial infarction risk, affected basically in part by the COX2 power inhibition; although all the NSAIDs except aspirin can be associated with a potential thrombotic risk. (3) **Renal events:** Renal toxicity is infrequent, but its risk is increased in those patients with renal or hepatic dysfunction, nephrotic syndrome, old age, diabetes, hypertension, cardiac congestive insufficiency or dehydration. (4) **Allergic Reactions:** Respiratory diseases exacerbation induced by NSAIDs, especially by non-specific NSAIDs are the most frequent hypersensitive drugs reactions. (5) **Haematological events:** Agranulocytosis and aplastic anemia are low frequency events. Due to the fact that agranulocytosis is a pharmacogenetic problem, local studies have to be made.

Some additional **myths and realities:** (1) The selective COX2 are **not** more efficacious than non-selective NSAIDs. (2) intravenous or IM administration of NSAIDs do **not** produce less GI adverse events and are not more efficacious. (3) concomitant use of 2 or more NSAIDs does **not** increase their efficacy, but does increase their toxicity. (4) The COX2 inhibitors can be used in low CV risk patients for short periods of time. (5) Agranulocytosis due to dipyrone is uncommon in Latin America, Spain and other countries. According to Cochrane, a Mexican, Latin America, Spanish and Polish consensus, dipyrone is an efficient drug with a very favorable cost/benefit and risk/benefit profile.

## Complications of corticosteroid epidural injections

Elías Atencio, MD, FIPP

Attending Specialist, Department of Pain Medicine at the Caja de Seguro Social, Complejo Hospitalario Metropolitano, Panama City, Panama

The complications associated with the injection of corticosteroids are rare, but a recent report (2012) noted an increase in fungal infections in the US, reporting a total of 650 cases with 39 deaths associated with this type of infection. Apart from the toxic effects of the corticosteroids in the intrathecal space, they are few the serious complications reported from this technique. A recent retrospective study examined a total of 4265 epidural corticosteroid injections in 1857 patients along 7 years. There were 161 cervical interlaminar injections, 123 lumbar, 17 caudal and 3964 transforaminal injections. No serious complications were identified, 103 minor complications (2.4%) were reported, including increased pain (1.1%), pain in the puncture site (0.33%), persistent heaviness (0.14%) and others (0.8%). The complications were less common with the transforaminal injections (2.1%) than with the interlaminar injections (6%). **In general overall complications can be grouped as follows:** (1) Neurotoxicity—arachnoiditis and aseptic meningitis due to intrathecal injection. The first is expressed as constant and burning low back and leg pain, increased urinary frequency and incontinence, muscle spasms in the back and legs, variable sensory disturbances and motor dysfunction. The second is usually a benign condition that causes signs of neurological irritation including burning pain in the legs, headache, meningismus and in severe cases seizures. It is difficult to determine which component of the steroid is the neurotoxic agent, Nelson suggests that polyethylene glycol is the causative agent. There is no definitive treatment for arachnoiditis or for aseptic meningitis; symptomatic treatment is the mainstay. (2) Other potential damages include cord injury by the injection needle, injection into a spinal artery with embolization. (3) Pharmacological effects such as cushingoid effects and impaired glucose tolerance.

## Anatomy of the trigeminal system, trigeminal neuralgia and its interventional treatment

Fabián C. Piedimonte

President in the CENIT Foundation for Neuroscience Research; and School of Medicine, Buenos Aires University, Buenos Aires, Argentina

The revised international classification of headache suggests 3 variants of trigeminal neuralgia (TN): (1) classic or idiopathic trigeminal neuralgia; (2) trigeminal neuralgia with persistent concomitant facial pain; and (3) symptomatic trigeminal neuralgia, caused by a distinct structural lesion other that vascular compression. Nevertheless, in a clinical aspect, the proposal by Burchiel has a wider and more comprehensive sense, differentiating the trigeminal pain in 7 subtypes based on their characteristics and causes. In terms of its pathophysiology, current opinion suggests that the classic TN is caused by a proximal compression of the trigeminal nerve root close to the brainstem through a tortuous blood vessel (an artery or a vein), leading to a process of secondary demyelination probably mediated by ischemic damage by microvasculature impairment. TN is an infrequent disease; analysis of some of the available studies suggest that the prevalence in the general population ranges between 0.01% and 0.3%, and the gender ratio of women to men is about 3:2. It can occur at any age, but over 90% of the cases occur above age 40, being its peak of onset between 50 and 60 years old. The incidence of TN in multiple sclerosis is higher than in the general population. Pain is unilateral, with only 3% of bilateral involvement; V3 and V2 branches are the most commonly affected individually or combined, while V1 is rarely affected. The medical treatment is based on the use of antiepileptic drugs (AEDs). The first-line therapy, according to current treatment guidelines based on evidence, should be carbamazepine (200-1200 mg/d) and oxcarbazepine (600-1800 mg/d). The second-line treatment, with less evidence, consists of adding lamotrigine (400 mg/d) or other anti-epileptics and baclofen. Failure of medical treatment should lead to surgical procedures such as (1) microvascular decompression, (2) percutaneous damage to the Gesserian ganglion by injecting drugs (glycerol), thermocoagulation by radiofrequency, or balloon compression, and (3) radiosurgery, by Gamma Knife.

## Monetary cost associated to pharmacological treatment for patients with chronic non-oncological pain in Mexico

Alfredo Covarrubias-Gómez^a^, Rodrigo A. Pavón-Sánchez^a^, Susana Ruiz-Ramírez^a^, Ana L. Garduño-López^b^

*Departments of* ^*a*^*Pain and Palliative Medicine, and* ^*b*^*Anesthesiology at Instituto Nacional de Ciencias Médicas y Nutrición Salvador Zubirán (Mexican NIH)*

**Objective:** To evaluate monetary cost of drug therapy in patients with chronic non-cancer pain in a Mexican Pain Clinic.

**Methods:** A retrospective, descriptive, and ethics committee approved research study. Those records that met inclusion criteria were selected. Related information was collected with the variables: pain (chronicity, type and intensity), therapeutic overall (total number of drugs ingested by the patient for diseases and cost associated with analgesic treatment), number of comorbidities and other of demographic type (age, gender and education). Descriptive statistics (measures of central tendency and dispersion) and inferential (correlation) statistics were applied.

**Results:** Twenty-two cases were analyzed. Sixteen women (73%) were identified. The average age was 61.82 years. Patients had a median of 5 diseases (mean number of prescribed medications was 8 and mean number of prescribed analgesics was 3). Twenty cases had chronic pain (91%). From those 8 cases had somatic pain (36.4%), 2 cases visceral pain (9%), 2 cases neuropathic pain (9%), 10 cases had both somatic and neuropathic (45.4%). Average monthly monetary cost of analgesic drugs for the treatment of chronic non-oncological pain was 1637.80 pesos (approx. 86.5 USD) (DS: 1095.14, minimum 286.20 y maximum 4242.00 pesos). Neuropathic plus somatic pain was the most expensive (mean 2170.7 pesos [114.24 USD], DS: 1282.72, minimum 673.2 y maximum 4242 pesos).

**Conclusion:** This study identified that higher pain intensity correlated with higher monetary cost. It was also noted that depending on the type of pain the cost is modified, a fact that could impact negatively on adherence to therapy. More studies are needed to fully understand the real impact of costs in socialized and private Latin-American medicine.

## Cancer pain management: pharmacological optimization

Patricia Gómez

Department of Anaesthesia and pain, Colombian National University

Cancer is one of the main causes for mortality in the world and cancer cases are expected to rise by 70% over the next 20 years. The World Health Organization (WHO)'s pain ladder, relieves pain in 60% to 80% patients, but has limitations due to the fact that nowadays cancer patients have a higher probability to survive and complications are mainly caused by neuropathic pain. The management of pain based on opioids, has caused concern over the past years, due to the rise in addiction to formulated opioids. This study included a PubMed search for keywords: cancer pharmacological management or treatment, over the last 10 years. Results indicate that the optimization of cancer pain management has to be based on pain mechanism that involves inflammation, neuropathic, ischemic, and compressive components. Pharmacological Management: The development of modern pharmacology has grown with the therapeutic targets due to peripheral and central sensitization studies, as well as the rise in the knowledge of the descending inhibitory system. (1) Opioids. Morphine continues to be the most used opioid, as its offers multiples advantages such as analgesic efficiency, different administration routes and low cost (Level 1a, 2011). Oxycodone and hydromorphone are useful drugs too. (2) Weak opioids (eg, codeine, tramadol) can be used only if pain is moderate, because they have a maximum recommended dose after which the adverse effects increase more than the analgesic effect. Due to problems caused by their metabolism, its used is not recommended for children and for 10% of older patients. (3) Tramadol and tapentadol have moderate evidences when it comes to neuropathic pain (Level 1a). (4) Fentanyl is useful in breakthrough pain. In addition, there are Nonopioides analgesics, Complementary or adjuvant drugs, and Non analgesics drugs such as biphosfanates. Adverse effects of these drugs, including nausea, emesis and constipation must be considered. In conclusion, the pain ladder is more suitable for patients that are at the end of their life. It is a mistake to treat chronic pain as if it was acute or end life pain. The goal is to decrease opioids and their adverse events doing a mechanism pain analgesic treatment, using multimodal analgesia, interventions and non-pharmacological management.

